# Therapeutic Effect of Carvacrol-loaded Albumin Nanoparticles on Arthritic Rats

**DOI:** 10.22037/ijpr.2019.15494.13131

**Published:** 2020

**Authors:** Nasser Gholijani, Samira-Sadat Abolmaali, Kurosh Kalantar, Mohammad-Hadi Ravanrooy

**Affiliations:** a *Autoimmune Diseases Research Center, Shiraz University of Medical Sciences, Shiraz, Iran. *; b *Department of Pharmaceutics, School of Pharmacy, Shiraz University of Medical Sciences, Shiraz, Iran. *; c *Department of Immunology, School of Medical, Shiraz University of Medical Sciences, Shiraz, Iran. *; d *Department of Chemistry, Payame Noor University, Shiraz, Iran.*

**Keywords:** Carvacrol, Albumin nanoparticles, Adjuvant-induced arthritis, Anti-inflammatory effect, Rheumatoid arthritis

## Abstract

Rheumatoid arthritis (RA) is one of the most common autoimmune diseases. Carvacrol, an important natural terpenoid product in aromatic plants such as *thyme*, has shown anti-inflammatory effects in animal models of arthritis. However, its poor water solubility and high volatility have limited its application. In the present study in order to overcome this problem, we encapsulated carvacrol in the bovine serum albumin (BSA) nanoparticles and examined its therapeutic and immunomodulatory effects in adjuvant-induced arthritis (AIA). Carvacrol-loaded BSA nanoparticles were prepared by desolvation method. Nanoparticles had encapsulation efficiency (EE) of 67.7 ± 6.9% and loading capacity (LC) of 26.6 ± 2%. The size of particles was 148 ± 25 nm and they had monomodal distribution. After arthritis induction, the rats were treated intraperitoneally with nanoparticle for every 3 days until day 28. The treatment of the rats with 375 mg/mL carvacrol-loaded BSA nanoparticle significantly decreased clinical severity score (27.5 ± 9.8%, *p* = 0.008), erythrocyte sedimentation rate (33.4 ± 10%, *p* = 0.02), nitric oxide production (82.3 ± 2.6%, *p* = 0.004) and interleukin (IL)-17 gene expression (55.1 ± 8.2%, *p* = 0.003) compared to the untreated arthritic group. A higher reduction in inflammation severity in arthritic rats treated with carvacrol-loaded BSA in comparison to those treated with carvacrol alone was observed. In conclusion, encapsulation of carvacrol in nanoparticles reduced arthritis signs and release of NO and IL-17 inflammatory cytokine and therefore is suggested to be considered as a good approach for improving the therapeutic applications of carvacrol in RA.

## Introduction

Rheumatoid arthritis (RA) is a chronic systemic inflammatory disorder and one of the most common autoimmune diseases, affecting around 1% of the world’s population (1). Synovial inflammation in RA leads to formation of rheumatoid pannus and joint destruction in response to a number of pro-inflammatory cytokines and tissue-destructive enzymes (2-3). Numerous studies have shown that pro-inflammatory cytokines such as IL-1β and TNF-α play critical roles in the generation of inflammatory and destructive responses in RA (4-5). Although RA is usually considered to be a Th1 mediated disease; attention has progressively more focused on the role of Th17 cells (6-7). Th17 cells infiltration have been detected in both synovial fluids and synovial membranes of RA patients (8-9). Adjuvant-induced arthritis (AIA), is an experimental model of RA which is extensively used for preclinical evaluation of many anti-arthritic components (10). 

Carvacrol, an important natural terpenoid product in aromatic plants such as *thyme*, has broad antimicrobial, antioxidant, and anti-inflammatory effects (11). There are a number of reports about inhibitory effects of carvacrol on inflammatory (IL1-β and TNF-α), Th1 (IL-2 and IFN-γ) and Th17 (IL-17) cytokines (11-13). Carvacrol has poor water solubility and is chemically unstable, and this restricts its therapeutics applications. Nanoencapsulation of carvacrol is suggested to increase the solubility of this compound (14-15).

Albumin is commonly used to prepare nanocapsules and nanospheres (16). This biopolymeric nanoparticle is water soluble, non-toxic, not harmful to living tissues, biodegradable and non-immunogenic. Carvacrol-loaded bovine serum albumin (BSA) nanoparticle can be prepared by desolvation method (17-18). This method is efficient for entrapment of natural components and the produced nanoparticles are spherical and stable, and release drug in a controlled and sustainable mode into the solution (19). Therefore, employing these nanoparticles may be considered as a novel approach for increasing the solubility of carvacrol and improving the therapeutic use of carvacrol in inflammatory-mediated diseases. 

In the present study in order to find the therapeutic and immunomodulatory effectiveness of carvacrol-loaded BSA nanoparticles, we prepared the nanoparticles by desolvation method and examined their effects on amelioration of AIA in rats. Also, the release and expression of various inflammatory mediators and cytokines in sera and tissues of the treated rats were investigated. 

## Experimental

*Preparation and optimization of carvacrol-loaded BSA nanoparticle*

BSA nanoparticles were prepared by desolvation method (17-18). Briefly, after dissolving 50 mg of BSA in 1 ml of distilled water and adjustment of pH to 8.5, tween 80 (0.5%) was added to the solution and stirred at 500 rpm for 30 min. Twenty-five milligrams of carvacrol were dissolved in 4 mL of ethanol and gradually dropped into the aforementioned solutions. After the desolvation process, for particle cross-linking, 12 μL of 8% aqueous glutaraldehyde solution was added and stirred at 500 rpm at room temperature for 24 h. The nanoparticles were purified by centrifugation at 9,000 rpm for 30 min at 25 °C and the supernatant collected. The free BSA was measured in the supernatant by BCA (Bicinchoninic acid) protein assay method (Thermo scientific, USA). After ethanol precipitation of albumin, an ultraviolet (UV) spectrophotometer (Biochrom WPA Biowave II, UK) was used to measure the free carvacrol level in the supernatant. The absorbance of the supernatant was determined at 275 nm (carvacrol maximum absorbance) (20). 

Particle size, zeta potential, and morphology of the nanoparticles were assessed using size analyzer, zeta analyzer, and atomic-force microscopy (AFM), respectively. The nanoparticles were dispersed in distilled water (pH 7.4) and their average size and zeta potential was evaluated by dynamic light scattering (DLS) (NANO-flex, Microtrac) and zeta analyzer (ZETA-check, Microtrac) instruments, respectively. A drop of nanoparticle sample was deposited on a slide, air dried and then scanned with atomic force microscopy (Agilent Technologies, Model 5500, Keysight technologies) for morphology and topography assessment of the nanoparticles. Measurements were performed in contact mode and CSE17 cantilever was used. 

The encapsulation efficiency (EE), loading capacity (LC), and nanoparticles yield (Y_np_) were calculated by the following Equations (17, 21):

$\mathrm{EE}=\frac{\mathrm{Total carvacrol}–\mathrm{Free carvacrol}}{\mathrm{Total carvacrol}}\times 100$

$\mathrm{LC}=\frac{\mathrm{Total carvacrol}–\mathrm{Free carvacrol}}{\mathrm{Nanoparticle weight}}\times 100$

$\mathrm{Ynp}=\frac{\mathrm{Total BSA}–\mathrm{Free BSA}}{\mathrm{Total BSA}}\times 100$

*Arthritis induction and treatments*

Thirty-five female Sprague Dawley rats (180-200 g) were kept under standard conditions. The rats were divided into five groups; each consists of seven animals. AIA was induced by a single subcutaneous injection of complete Freund’s adjuvant (CFA) containing 10 mg/mL of heat-killed Mycobacterium (H37 Ra) (0.1 mL) at the base of the rat tails, according to the previous studies (22-24). 

A group of rats were injected with normal saline and received PBS intraperitoneally (IP) (normal)*.* The arthritic groups were treated IP with 50 µL of a) olive oil as vehicle (untreated group), b) one-hundred milligram carvacrol/mL olive oil, c) two-hundred seventy-five milligram BSA nanoparticle/mL olive oil and d) three-hundred seventy-five milligram carvacrol-loaded BSA nanoparticle/mL olive oil every 3 days from the beginning of the arthritis induction. The rats were sacrificed on day 28 of arthritis induction and sera, lymph nodes and inflamed tissues were collected for further experiments.

*AIA clinical score and body weight monitoring*

The clinical signs and weight of the rats in each group were assessed daily. An arthritis scoring system with maximum score of 16 was used to determine the severity of arthritis as the following. The level of arthritic inflammation of each paw was graded from 0 to 4 based on degree of erythema, swelling and joints deformation: 0 = normal; 1 = slight swelling or erythema of one toe or finger; 2 = swelling and erythema of two toes or fingers; 3 = severe swelling and erythema of the wrist or ankle; 4 = complete swelling and erythema of toes or fingers, joint deformity and lack of flexibility. 

*Erythrocyte sedimentation rate (ESR) evaluation*

The level of ESR as an indicative of inflammation was determined by capillary tube test (micro-ESR). Briefly heparinized capillary tubes were filled with the citrated venous blood up to the 10-cm mark (0.2 mL of blood). The tubes were kept vertical and read after 1 h for the sedimented red cell level.

*Determination of nitric oxide (NO) concentration*

NO production was measured using the colorimetric method of Griess in inflamed tissue extracts (25). Eighty microliters of the tissue extracts were mixed with an equal volume of Griess reagent and incubated for 10 min at room temperature. The OD of reaction product was measured at 550 nm and nitrite concentrations calculated by comparing the OD values for the test samples to a standard curve generated by serial dilution of sodium nitrite.

*Real-time PCR analysis of inflammatory cytokines*

RNA from the inguinal lymph node cells was prepared using Parstous RNA extraction kit according to manufacturer instructions. Concentration and purity of RNA samples were checked using a Picodrop system (Picodrop, Hinxton, UK) and gel electrophoresis. Then, 10 μL of RNA from each condition were reverse-transcribed to cDNA using high-capacity cDNA reverse transcription kit in the presence of reverse transcriptase, dNTP mix, and random hexamer at 37 °C for 120 min. For analysis of TNF-α and IL-1β, real-time PCR was performed in a final volume of 20 μL containing 2 μL cDNA, 10 μL SYBR Premix Ex Taq II, 0.8 μL forward primer (10 pM), 0.8 μL reverse primer (10 pM), 0.4 μL ROX reference dye2, and 6 μL double-distilled water. The primers used were: β-actin [forward], 5-GCAAATGCTTCTAGGCGGAC-3; β-actin [reverse], 5-AAGAAAGGGTGTAAAA-CGCAGC-3; TNFα [forward], 5-TCAGCCTCTTCTCAT-TCCTGC-3; TNFα [reverse], 5-TTGGTGGTTTGCTACGACGTG-3; IL-1β [forward],50-GACAGAACATAAGCCAAC-3; and, IL-1β [reverse], 5-CACAGGACAGGTATAGAT-3; IL-17 [forward], 5-CTACCTCAACCGTTCCACTT-3; and, IL-17 [reverse], 5-ACTTCTCAGGCTCCCTCTTC-3. Real-time PCR was performed in an Applied Biosystems StepOne system (Foster City, CA). PCR conditions were as follows: one cycle at 95 °C for 30 sec, followed by 40 cycles at 95 °C for five sec, 59 °C for 18 sec, and 72 °C for 30 sec. β-actin served as an endogenous control and results of target mRNA levels were normalized against β-actin mRNA in each sample.

*Cytokine assay*

The levels of TNF-α and IL-1β cytokines in the rats’ sera and inflamed tissue extracts were measured by enzyme-linked immunosorbent assay (ELISA) kits with the sensitivity of 16 and 39 pg/mL, respectively (eBioscience, USA). The collected inflamed tissues were weighed and immediately homogenized using a TissueLyser device (Qiagen, Hilden, Germany) in 1 mL of ice-cold homogenization buffer (Tris, 10 mol/L; NaCl, 50 mmol/L; MgCl_2, _2.5 mmol/L, pH 7.4) containing protease inhibitor (PMSF, 1 mM). The tissue homogenates were centrifuged at 15000 × g for 40 min at 4 °C, and then the supernatant was collected and stored at −80 °C until used. ELISA assay was performed as described by the manufacturer. Briefly, 96-well micro plates were coated an overnight with 100 μL capture antibody at 4 °C. The plates were blocked, and different standards and samples (100 μL/well) were added to the appropriate wells and incubated an overnight at 4 °C. Kit-provided detection antibody (100 μL) was added to wells and the plate incubated for 1 h at room temperature. After washing, 100 μL of streptavidin-HRP (30 min), 100 μL of tetramethylbenzidine (TMB) substrate (15 min), and then 50 μL of stop solution (1 M phosphoric acid) was added. The absorbance of reaction was measured using a microplate reader (Biotek, Nevada, USA) at 450 nm with a background subtraction at 570 nm.

*Statistical analysis*

The data were expressed as mean ± SD unless otherwise specified. Significant differences between the groups were evaluated using Prism software (GraphPad, San Diego, USA) containing appropriate statistical tests *e.g.*, one-way ANOVA, and a Student’s *t*-test. A *p*-value < 0.05 was considered significant.

## Results

*Morphology, size and zeta potential analysis of carvacrol-loaded BSA nanoparticle*

**Figure 1:**
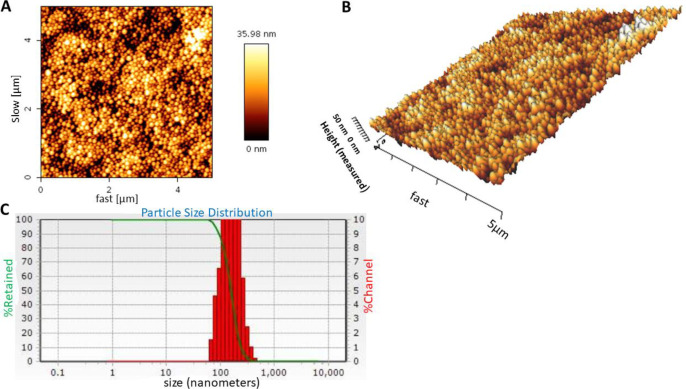
(A and B) Atomic-force microscopy (AFM) image and (C) dynamic light scattering (DLS) histogram of carvacrol-loaded nanoparticles. The loading was performed with the weight ratio of 2:1 (BSA to carvacrol). AFM and DLS results of the nanoparticles were evaluated in three independent experiments

Three-dimensional shape (topography) of carvacrol-loaded BSA nanoparticles was examined with AFM. As shown in Figures 1A and 1B, the nanoparticles were spherical with a smooth surface and homogeneous size distribution. The mean size of nanoparticles was 148 ± 25 nm (n = 3) with polydispersity index (PDI) of 0.32 ± 0.1 (Figure 1C), which indicated that the size distribution was homogeneous and monomodal. Zeta potential of nanoparticles was -10.66 ± 1.5 mV, indicating colloidal stability of the nanoparticles due to their negative surface charges.

*Encapsulation efficiency (EE), loading capacity (LC) and nanoparticles yield (Y*_np_ *)*

The percentage of EE and LC of the nanoparticles were 67.7 ± 6.9 and 26.6 ± 2.0, respectively which showed a high-affinity binding of albumin to carvacrol. Y_np_ of nanoparticles was 92.9 ± 0.6%. 

*Effects of treatments on arthritis score and weight of animals*

**Figure 2:**
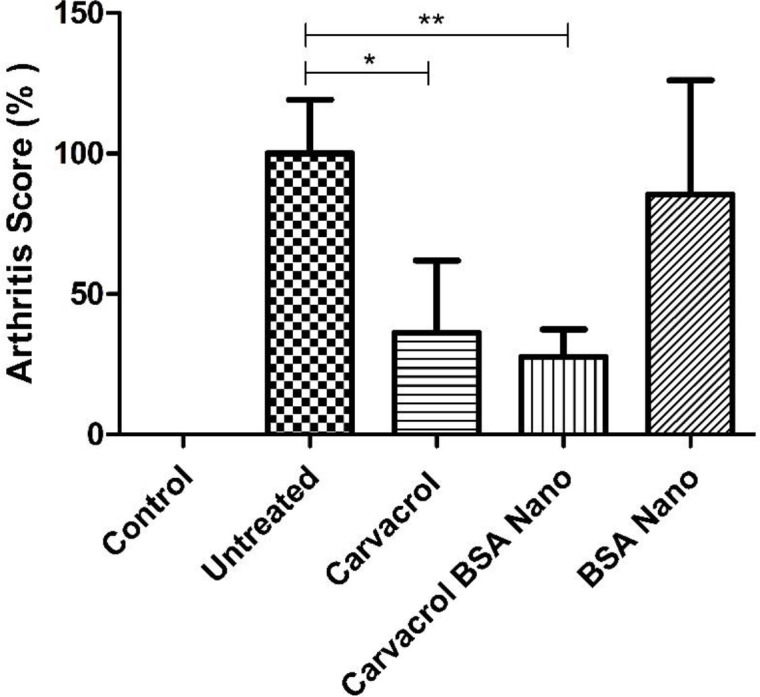
Effects of carvacrol-loaded nanoparticles on arthritis score. Rats were divided in five groups, control and four adjuvant induced arthritis groups treated either with the vehicle (untreated), carvacrol alone, carvacrol-loaded BSA nanoparticles and BSA nanoparticles. Data shown are means ± SE (n = 7/group). Values are significantly different from untreated rats at ^*^p < 0.05; ^**^p < 0.01

Treatment with carvacrol alone and carvacrol-loaded BSA nanoparticles significantly decreased clinical score to 36.1 ± 25.8% (*p* = 0.04) and 27.5 ± 9.8% (*p* = 0.008) of the untreated group, respectively (Figure 2). 

*Effect of treatments on ESR*

**Figure 3:**
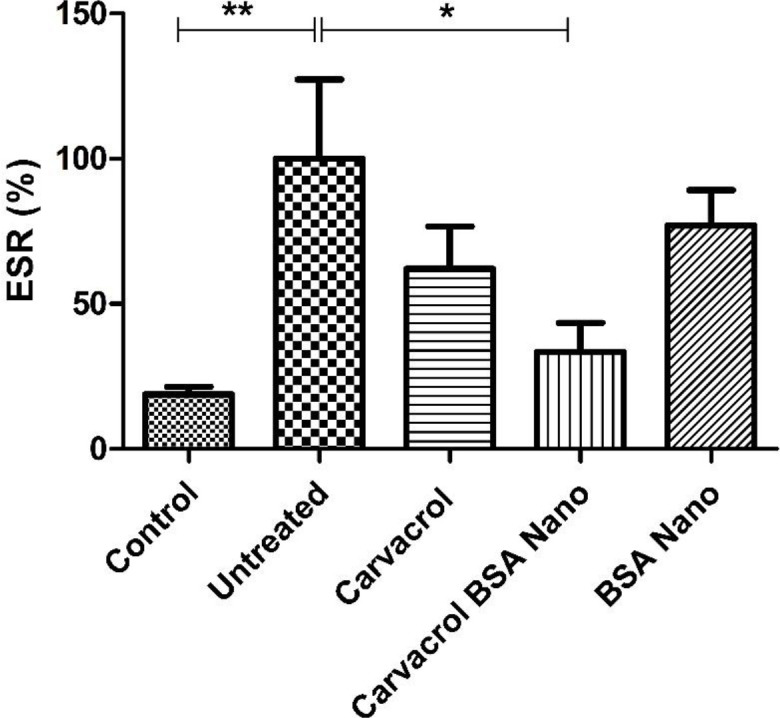
Effects of carvacrol-loaded nanoparticles on erythrocyte sedimentation rate (ESR). Rats were sacrificed in day 28 and blood samples collected. ESR was performed by capillary tube. Values shown are means ± SE (n = 7/group). ^*^p < 0.05; ^**^p < 0.01 show significant results

As shown in Figure 3, the induction of arthritis in rats by mycobacterial adjuvant, significantly increased ESR from 18.8 ± 2.6% in control rats to 100 ± 27.3% in arthritic rats (*p* = 0.002). Carvacrol-loaded nanoparticles significantly reduced this value to 33.4 ± 10% (*p* = 0.02). The treatment of the rats with carvacrol alone reduced this value to 62 ± 14.6%, but the result was not significant.

*Effect of treatment on NO production*

**Figure 4:**
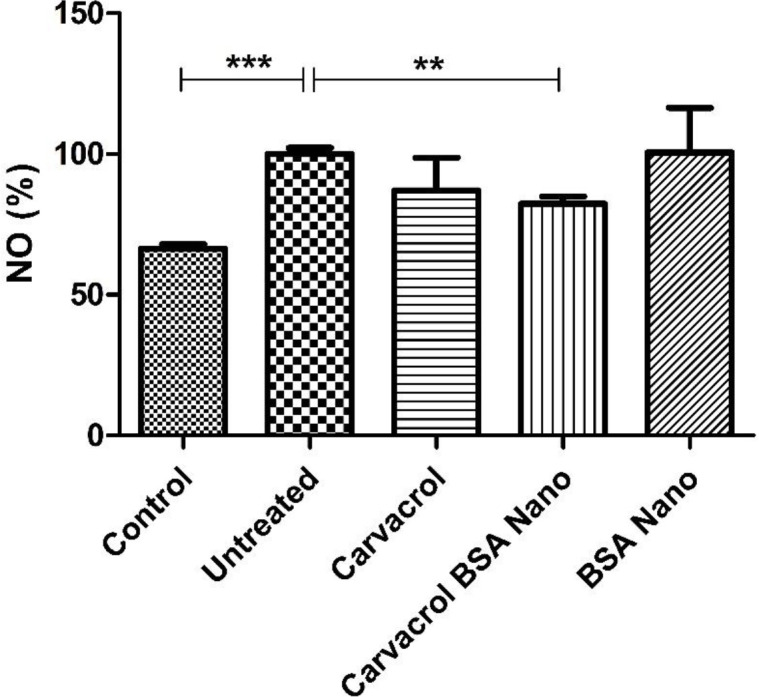
Effects of carvacrol-loaded nanoparticles on tissue NO production. Rats were sacrificed in day 28 and inflamed paw tissue extracts prepared. NO level was evaluated by Griess reagent. Values shown are means ± SE (n = 7/group). ^***^p < 0.001, ^**^p < 0.01 shows significant result

NO production in the inflamed tissue extracts was measured using Griess reagent. As shown in Figure 4, adjuvant-induced arthritis in the rats increased NO production from 66.3 ± 1.7 in control rats to 100 ± 2.2% (*p* = 0.0003). Carvacrol-loaded BSA nanoparticles significantly decreased this value to 82.3 ± 2.6% (*p* = 0.004). 

*Effects of treatment on inflammatory cytokines genes’ expression and production*

**Figure 5:**
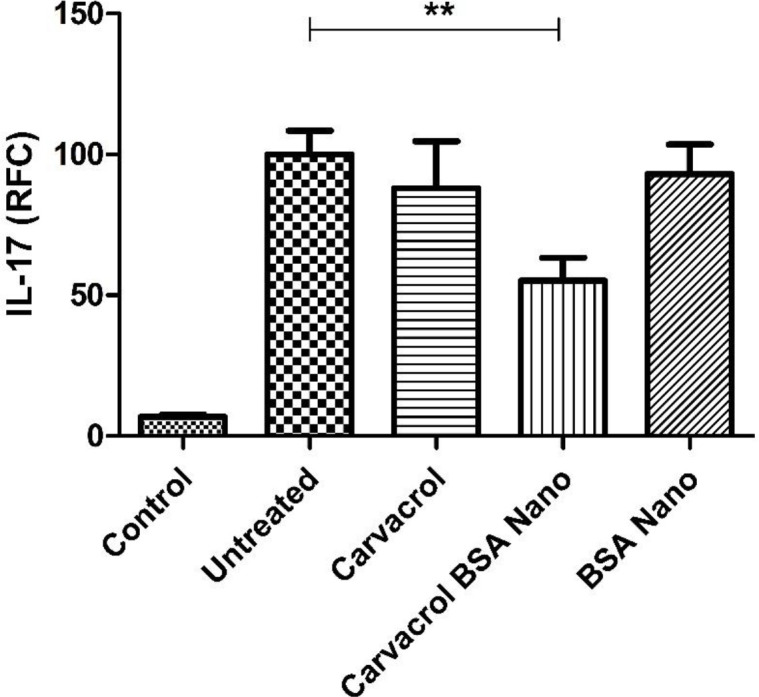
Effects of carvacrol-loaded nanoparticle on IL-17 gene expression in lymph node cells. Isolated lymphoid cells were examined for IL-17 gene expression by real time PCR. Values shown are means ± SE (n = 7/group). ^**^p < 0.01 shows significant result

IL-1β and TNF-α cytokine levels were measured in sera and tissue extracts of the inflamed paw by ELISA method. There were no significant differences in the level of sera and tissue extracts of these cytokines between the groups. Also the levels of IL-1β, TNF-α, and IL-17 genes expression were evaluated by real-time PCR on the isolated lymph node cells. Induction of arthritis in the rats increased the level of IL-17 gene expression from 6.8 ± 0.9% in the control rats to 100 ± 8.5% in the arthritic rats (*p* = 0.005). Carvacrol-loaded nanoparticles significantly decreased this value to 55.1 ± 8.2% (*p* = 0.003) (Figure 5). There were no significant differences in the level of IL-1β and TNF-α genes’ expression between the groups (data not shown).

## Discussion

The aim of the current study was to evaluate the effects of carvacrol-loaded BSA nanoparticle on amelioration of joint damage in AIA as a popular animal model of RA. 

Carvacrol is an anti-inflammatory agent with poor water solubility and chemical instability. There are various methods to increase the solubility and stability of the natural products including nanoparticle coating, semisolid preparations, water-soluble cellulose derivative coating, and chelating agents (26). In few studies, the researchers have tried to overcome this disadvantage of carvacrol. Albumin is easy injectable and can be used as a suitable carrier for drug delivery (27). Moreover, this molecule has several reactive groups on its surface for covalent attachment of the components. We encapsulated carvacrol in albumin nanoparticle to promote its stability and effectiveness. Topography of carvacrol-loaded BSA nanodrug showed the nanoparticles with spherical shape, smooth surface, and homogeneous size distribution. The mean size of nanoparticles was approximately 148 nm and the polydispersity index (PDI) was around 0.32 which indicated that the size distribution of the nanoparticles was homogeneous and the monomodal distribution has been formed. Near 67% efficiency of encapsulation and 26% of the loading capacity of nanoparticles showed high-affinity binding of albumin protein to carvacrol (or vice versa).

As our results demonstrated, carvacrol-loaded BSA had a significant reducing effect on clinical score (arthritis severity) in the treated rats (≈27.5% of untreated rats). This decreased level in addition to the reduced ESR (≈33.4% of untreated rats) was observed in the treated rats indicated the efficiency of the carvacrol BSA encapsulation method to ameliorate arthritis. Treatments of AIA rats with carvacrol alone also significantly decreased the severity of arthritis to near 36% of the untreated group. Comparison of the treatment groups showed a higher reduction in inflammation severity in AIA rats treated with carvacrol-loaded BSA than those treated only with carvacrol. From these results, it can be concluded that the entrapment of carvacrol in albumin nanocapsules increases the carvacrol efficacy.

Various types of immune cells are detectable in the RA synovium. These are CD4+ T cells, CD8+ T cells, B cells, NK cells, γδ T cells, macrophages, mast cells, and myeloid cells. A range of soluble mediators such as NO, TNF-α, IL-1β, and IL-17A that are produced by these cells have been shown to correlate with disease severity and/or progression (6, 28-30). In this study, we observed an approximately 18% reduction in NO production after treatment of the rats with carvacrol-loaded nanoparticles compared to the untreated group. In contrast, we did not find any significant decline in gene expression levels of IL-1β and TNF-α cytokines in inguinal lymph node cells. The level of these cytokines in sera and inflamed tissue extracts were not also different between the groups. Because the arthritis signs in AIA model begin to decline on day 28 after disease induction, it is probable that the levels of these innate immunity cytokines have decreased to normal levels. 

We showed that the treatment of arthritic rats with carvacrol-loaded nanoparticles, in contrast to carvacrol alone, could down-regulate IL-17 gene expression level to near half of the level in untreated group. Several studies have emphasized the importance of Th17 and its main cytokine, IL-17 in RA development, progression, and diseases severity (31-33). These results supported the more efficiency of the carvacrol nanoparticles than carvacrol alone in reducing inflammatory response and also suggested the possibility that carvacrol-loaded BSA, in part by down-regulation of Th17 responses was contributed to amelioration of AIA. In conclusion, carvacrol-loaded BSA nanoparticles prepared by desolvation method were spherical and stable. As results of this study showed, these nanoparticles could ameliorate AIA and reduce inflammatory signs in arthritic animals more efficiently than carvacrol alone. Moreover, the carvacrol-loaded BSA nanoparticles decreased the release of NO and IL-17 inflammatory cytokine in the treated animals. Therefore, encapsulation of carvacrol in BSA is suggested to be considered as a good approach for improving the therapeutic applications of carvacrol.
